# Work Function Engineering of Graphene

**DOI:** 10.3390/nano4020267

**Published:** 2014-04-03

**Authors:** Rajni Garg, Naba K. Dutta, Namita Roy Choudhury

**Affiliations:** Ian Wark Research Institute, University of South Australia, Mawson Lakes Campus, 5095 Adelaide, Australia; E-Mails: garry014@mymail.unisa.edu.au (R.G.); namita.choudhury@unisa.edu.au (N.R.C.)

**Keywords:** graphene, graphene oxide (GO), reduced GO (RGO), functionality, bandgap, work function (WF), high occupied molecular orbital (HOMO), lower occupied molecular orbital (LUMO), hole transporting layer (HTL)

## Abstract

Graphene is a two dimensional one atom thick allotrope of carbon that displays unusual crystal structure, electronic characteristics, charge transport behavior, optical clarity, physical & mechanical properties, thermal conductivity and much more that is yet to be discovered. Consequently, it has generated unprecedented excitement in the scientific community; and is of great interest to wide ranging industries including semiconductor, optoelectronics and printed electronics. Graphene is considered to be a next-generation conducting material with a remarkable band-gap structure, and has the potential to replace traditional electrode materials in optoelectronic devices. It has also been identified as one of the most promising materials for post-silicon electronics. For many such applications, modulation of the electrical and optical properties, together with tuning the band gap and the resulting work function of zero band gap graphene are critical in achieving the desired properties and outcome. In understanding the importance, a number of strategies including various functionalization, doping and hybridization have recently been identified and explored to successfully alter the work function of graphene. In this review we primarily highlight the different ways of surface modification, which have been used to specifically modify the band gap of graphene and its work function. This article focuses on the most recent perspectives, current trends and gives some indication of future challenges and possibilities.

## 1. Scope of the Review

**Figure 1:**
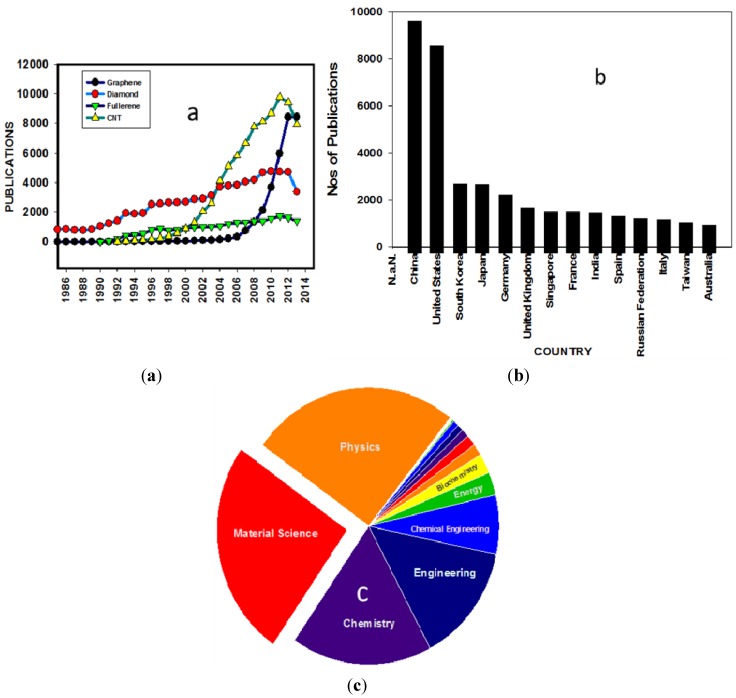
History of scientific publications on carbon-based materials: (**a**) Total annual number of publications for each carbon allotrope based on Scopus; (**b**) History of the number of publications in graphene from different countries; (**c**) History of the number of publications on grapheme based on different subject areas.

Graphite and its intercalation compounds have been studied for over 150 years; however, serious scientific investigation on graphene is of rather recent origin, from when Novoselov *et al.* [1] 2004 reported the facile synthesis of single layer/few layer graphene from graphite using mechanical exfoliation from graphite. Since this report on the unusual electronic properties of single layers of the graphene lattice, research interest on graphene increased exponentially over the past decade (Figure 1).

Andre Geim and Konstantin Novoselov share the 2010 Nobel Prize in Physics for their ground-breaking work on graphene. Figure 1a illustrates the annual number of articles published in refereed journals containing different carbon allotropes as key words. The recent dramatic growth of publications in graphene is remarkable with more than 8000 papers published in 2012 (~22 papers/day). Figure 1b shows that China and USA are the two countries that are pursuing research on graphene most vigorously (~25% of total publication each). Moreover, the aspects of graphene from physics and material science can be identified as the two major focused areas of research (Figure 1c). Although, a large number of review and prospective articles on different aspects of graphene are plentiful, there are however a limited number of critical reviews on graphene interfacial engineering and work function (WF) tuning. Most of the organic electronics, optoelectronics and printed electronics technologies require electrodes and conductors with an appropriately tuned work function to facilitate efficient charge transport. The band alignment of two different materials at the heterojunction is also governed by their respective WF. Therefore, in graphene-based electronic devices the WF of graphene under a given metal electrode is critical information for the realization of high-performance graphene-based interconnects. In this review, we focus our attention on the recent advances on different methods for graphene synthesis and modification and its effect on tuning of WF. Initially we discuss briefly the unique band structure and a short history of the synthesis and characteristics of graphene, as well as the exciting recent progresses.

## 2. Graphene: A Unique Carbon Allotrope

**Figure 2:**
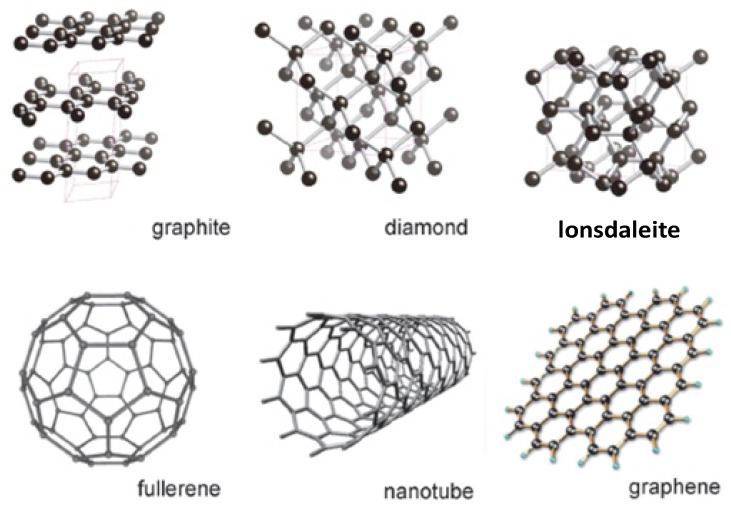
The common naturally-occurring sp^2^ and sp^3^ allotropes of carbon occur in different crystallographic forms. **Graphite**: Hexagonal; stacked flat layers of 3-coordinated sp^2^ C. **Diamond**: Cubic; framework of 4-coordinated sp^3^ C. **Lonsdaleite**: Hexagonal; framework of 4-coordinated sp^3^ C. **Fullerenes**: Closed cage molecules sp^2^ C: C60, C70, C76, *etc.* Nanotubes cylindrical fibers of sp^2^ C, single tubes or nested. **Graphene**: one-atom-thick graphitic layers with sp^2^ bonding. Reprinted with permission from [3]. Copyright 2013 Mineralogical Society of America.

The element carbon occurs in several allotropes, including crystalline, three-dimensional diamond, graphite and lonsdaleite; two dimensional graphene, one-dimensional nanotubes, zero dimensional fullerene (Figure 2) and several other non-crystalline forms [2,3]. These wide-ranging carbon allotropes exhibit extremes in the physical, chemical and morphological behaviour; which display the diversity of carbon’s atomic structures, crystal chemistry and bonding differences. For example, in diamond each C-atom is sp^3^ hybridized, the C–C–C bond angle is 109.5°, the C–C bond length is 1.54 Å; and forms basic tetrahedral units and a cubic unit cell. On the one hand diamond is considered as the hardest known substance. On the other hand, graphite is known to be one of the softest materials; and acts as an efficient solid lubricant. In graphite, graphene, fullerenes, carbon nanotubes, and several types of other amorphous and glassy carbons; the carbon atoms are in a planar three-coordination state that results from the sp^2^ hybridization. In this trigonal coordination state typical C–C distance is ~1.42 Å and C–C–C angles are ~120°. Graphene (GR) is a flat single layer, with sp^2^ hybridized carbon atoms tightly packed into a two dimensional honeycomb lattice structure; and it is one of the most exciting two dimensional materials being investigated today [4]. Graphene may also be considered as the basic building block for graphite materials of all other dimensions. It can be stacked into 3D graphite, rolled into 1D carbon nanotubes and wrapped into 1D fullerenes. The sp^2^ hybridisation forms strong directed bonds and determines a honeycomb lattice structure for graphene; and the p^z^ (π) orbitals form a delocalized π-system which regulates the conduction properties/charge mobility of graphite.

**Figure 3:**
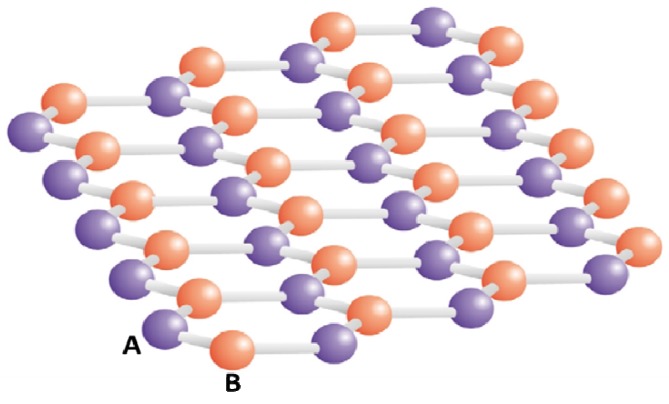
Schematic representation of the hexagonal arrangement of the carbon atoms in graphene. It can be reduced into two interpenetrating sub-lattices of carbon atoms with inversion symmetry between them. Atoms from different sub-lattices (**A** and **B**) are marked by different colors. Reprinted with permission from [6]*.* Copyright 2007 Elsevier.

Graphene is the first example of a close-packed two dimensional (2D) crystalline material isolated in nature; and it is currently receiving unusual growth in research attention. The hexagonal arrangement of the carbon atoms in graphene can be reduced into two interpenetrating sub-lattices of carbon atoms with inversion symmetry between them (Figure 3) [5].

## 3. Graphene: A Two Dimensional Form of Carbon with Unusual Band Structure and Characteristics

**Figure 4:**
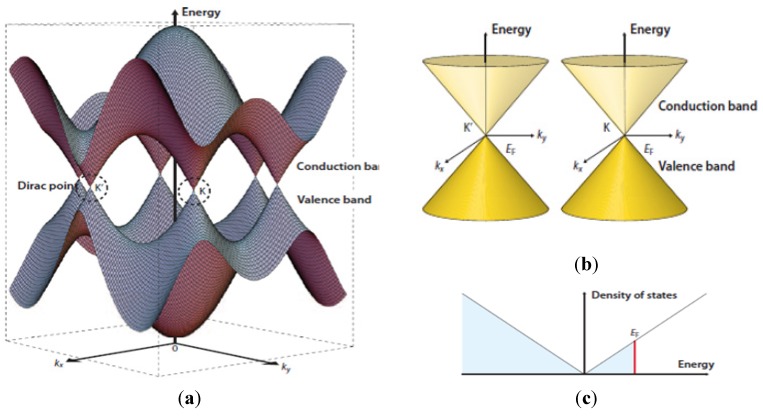
(**a**) Energy bands near the Fermi level in graphene. The first Brillouin zone of graphene is illustrated in the horizontal plane and labelled with some points of interest. The conduction and valence bands cross at points K and K'—the two non-equivalent corners of the zone, also known as the Dirac points; (**b**) Conic energy bands in the vicinity of the K and K' points; (**c**) Density of states near the Fermi level with Fermi energy *E*_F_. Reprinted with permission from [7]. Copyright 2009 Nature publishing group.

Graphene is a two-dimensional zero-band gap semiconductor with a unique energy diagram shown in Figure 4 [7]. This unique topology provides an unusual energy dispersion relation in graphene [8]. Graphene’s unique electronic structure is characterized by the pointed valence and conduction bands that meet at a single point in momentum space (the Dirac crossing energy). Quantum mechanical hopping between the sub-lattices in graphene leads to the formation of two energy bands, and their intersection near the edge of the Brillion zone yields the conical energy spectrum near the Dirac points K and K' as represented in Figure 4 [7]. It has now been confirmed that the charge carriers in graphene can be described by the Dirac-like equation, rather than the usual Schrödinger equation [6]. Novoselov *et al.* [1] demonstrated the strong ambipolar electric field effect of GR with electrons and holes in concentrations up to 10^13^/cm^2^, and with room temperature mobility of ~10,000 cm^2^/V∙s.

It has also been demonstrated that through selective control of the carrier concentration in the graphene layers, the band structure can be easily tuned near the Dirac crossing [9]. The electronic structure, unique morphological and electronic characteristics of graphene; and its potential applications have been the subject of extensive theoretical and experimental investigations [10,11,12,13]. Summarily, graphene has many special attributes including the followings unique characteristics:

(1) Graphene is a zero-band gap perfect 2D semi-conductor with a tiny overlap between valence and conduction bands [6];
(2) By strictly confining electrons to two dimensions graphene displays an unusual fractional quantum Hall effect [14,15,16,17];
(3) Graphene shows a strong ambipolar electric field effect with charge carrier concentrations up to 10^13^ cm^2^ and room temperature mobility of ~10,000 cm^2^·s^−1^ [6];
(4) Graphene demonstrates transport via relativistic Dirac Fermions [18,19];
(5) Graphene displays high thermal conductivity with a value of ~5000 Wmk^−1^ for a single layer sheet at room temperature [19];
(6) Graphene exhibits high optical transparency with absorption of ~2.3% towards visible light [20];
(7) Graphene is incredibly strong, mechanically (tensile strength of ~130 GPa, for a defect free single layer, Young’s modulus of 1 Tpa, third order elastic stiffness ≈2 Tpa); while remaining highly flexible and very light (0.77 mg m^−2^) and possesses a very high specific surface area (~2630 m^2^·g^−1^) [21].

## 4. Graphene: Potential Applications and Importance of Work Function

Despite its short history, graphene, graphene-related nanomaterials and their processing including their synthesis, direct liquid-phase exfoliation of graphite to produce single/few layered pristine graphene, doped graphene, graphene oxide, fluorographene, graphene, graphdiyne, and porous graphene have all been the subject of many recent reviews [22,23,24,25,26]. Allen *et al.* [27] presented a comprehensive review on graphene from the materials chemistry stand point. In-depth discussion focused on the physics aspect of graphene has been the subject of many recent perspectives and reviews [5,13,28,29]. The challenges and prospects of graphene based new energy materials specifically for supercapacitors, lithium ion batteries, water splitting, electrocatalysts for fuel cells, and solar cells have recently been reviewed by Sun *et al.* [30]. Recent advances in the field of graphene from the standpoint of electrochemistry have been presented by Chen *et al.* [31]. Some of the recent progresses in graphene synthesis, fundamental concepts and prominent applications in electronics and photonics have been summarized by Brownson *et al.* [32]. Compton and Nguyen [33] have highlighted the wide ranging functionalized materials that can be prepared using a bottom-up approach from bulk dispersions of graphene oxide, HRG, and graphene in various media. The opportunities and challenges of graphene-based hybrid materials for energy and sensing applications have been highlighted [34,35,36]. Graphene has also been promoted as a reinforcing filler in polymer composites and nano-composites, and is expected to be a less expensive substitute than carbon nanotubes [37]. In a recent review, Kuila *et al.* [38] discussed the different methods used for chemical functionalization of graphene and its importance in processing, properties and final applications. Yang *et al.* [39] reviewed the available different protocols for functionalization of graphene with special reference to its potential biomedical applications including drug delivery and multimodal imaging to preparation of bio-sensing devices. Craciun *et al.* [40] discussed the possibilities to engineer the electrical and optical properties in graphene through chemical functionalization of graphene. Such modifications have the potential to widen the applications of graphene devices on an industrial scale.

Due to its unusual electronic characteristics and distinct capabilities graphene has been used successfully to fabricate a number of simple electronic devices [10,41]. One of the major forces for the use of graphene in nanotechnology is to develop integrated circuits on a much smaller scale that is possible with current silicon-based complementary metal-oxide-semiconductor (CMOS) technology, and it has been identified as the most promising material for post-silicon electronics [42,43]. It has been predicted [44] that evolutionary miniaturization using silicon based technology will reach the fundamental limits of extreme physical size in the near future. Since graphene can exhibit room-temperature ballistic transport over mean free paths of up to 300 nm [6,45] its use has been suggested either as a channel material for the next generation of field-effect transistors (FET); or as a conductive sheet upon which nanometer scale devices may be patterned to create single electron or few electron transistors. Graphene is also an excellent candidate for ultra-high frequency transistors [26]. It has been identified as a novel electrode material with excellent stability, high transparency, flexibility and outstanding charge transfer mobility, which is a critical requirement for many optoelectronic devices such as: organic solar cells (OSCs), organic light-emitting diodes (OLEDs), and organic thin-film transistors (TFTs) [46,47,48,49,50]. The unique properties of graphene have also potential for use in gas sensors [51], supercapacitors [52] and printed electronics [35]. Recently, a CMOS compatible graphene photodetector covering all optical communication bands was also reported [53]. In all these practical device designs the WF of graphene is one of the most important considerations; moreover, WF is also dependent on the environment, such as adjacent substrates [54]. Another important consideration for both fundamental property measurements and practical device design is the contact resistance, and the choice of contact material. In graphene electronics, the contact resistance (*R*c) between graphene and other metal limits the performance of the device such as the carrier mobility and ON-state current of graphene field effect transistors (FETs) [55]. The contact resistance suppresses the on-current, which is detrimental to high-frequency transistor performance; however, in optoelectronics this effect enhances photocurrent efficiency [56]. A lower WF can dramatically enhance the emitting current [57]. The WF of graphene electrodes is also critical to maximize energy conversion efficiency in OPVs. Besides good conductivity and transparency of the electrode, the performance and current density for semiconducting electronic devices such as light-emitting diodes and field-effect transistors is strongly dependent on the carrier injection efficiency through the contact between electrodes and semiconducting material layers. Graphene being a prospective conductive material with the desirable properties including engineered WF for efficient carrier injection, makes it highly attractive. Due to the critical importance, different approaches have been investigated to modulate the WF of graphene including employing an external electric field [58], reaction with organic and inorganic molecules [59], chemical modification of the surface [60], metal doping [61,62,63], substrate orientation [64], and a self-assembled monolayer formation [65]. Intercalation of different species such as hydrogen [66], fluorine [67], lithium [68], gold [69] and iron (III) chloride [70] has also been identified as a potential method to modulate the work function of few layer graphene [71,72]. However, controlling the WF of graphene, precisely, on demand has yet to be demonstrated.

## 5. Graphene Synthesis: Relationship to Applications

Graphene was first successfully synthesized using mechanical exfoliation of graphite in 2004 by Novoselov *et al.* [1]. Such single/few layer graphene was used to elucidate the unique characteristics of graphene, which was the catalyst for the explosive growth in research on graphene. However, this method is unreliable, of low yield and basic; and it has very limited relevance to commercial high-end electronic applications. As the research interest and promise of large scale application of graphene has grown extensively, various practical methods have been attempted to synthesize high quality pristine graphene in large scale at low cost. The fundamental properties of graphene have been well investigated using high-quality graphene produced by “top-down” physical exfoliation [1,72] and solvation-assisted exfoliation of graphite [73]. Among the many approaches for single or few layer graphene synthesis, the most significant ones are the exfoliation of graphite [74,75,76,77], arc discharge of graphite in the presence of helium and hydrogen gases [78], laser based green synthesis, [79], chemical vapour deposition [35,80], low-cost liquid-phase exfoliation of graphite and graphene oxide reduction [81]. In Table 1, we summarize the synthesis methods employed; and comment on their maturity, advantages and disadvantages, and targeted use. Before the wider application of graphene, the most important challenge is to develop a facile and efficient method for controlled production of processable, large graphene sheets of the desirable properties for specific applications. An important challenge in the large-scale production of pristine graphene is to select/design the proper exfoliating reagents including the right solvents. The continuous and scalable large area synthesis of graphene by chemical vapour deposition (CVD) has reinforced its study in a broad range of research areas [82,83]. Recently, Colson *et al.* [84] demonstrated covalent organic framework thin films on single-layer graphene. The electronic characteristics of a planar covalent organic framework (COF) on graphene are investigated by means of dispersion-corrected density functional theory [85].

**Figure 5:**
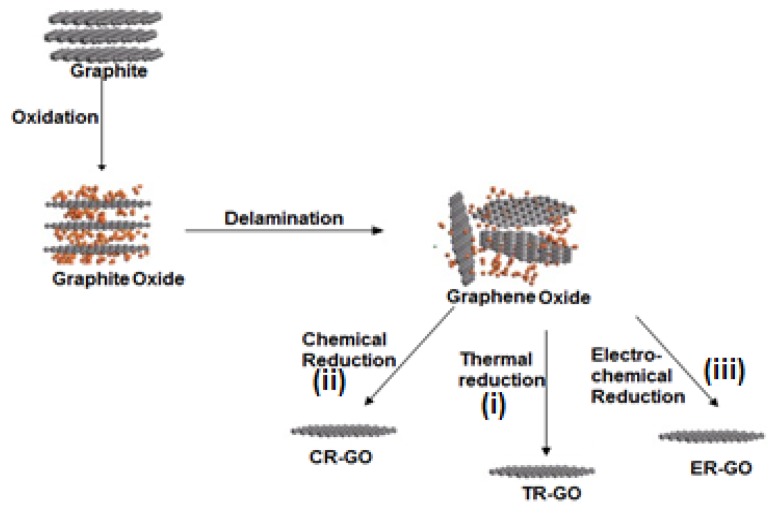
Schematic representation of the synthesis of single/few layer graphene from graphite: An oxidative treatment is initially performed to generate graphite oxide; which is followed by exfoliation to produce graphene oxide. Finally, (**i**) Thermal reduction; (**ii**) Chemical reduction or (**iii**) Electrochemical reduction of graphene oxide produces reduced graphene (r-GO).

Ideally, the exfoliation protocol should involve the use of low-cost natural graphite powder as starting material, with commonly available solvents to assist that can be removed easily to produce pristine graphene. Various suitable chemical methods have been developed and identified for mass production of graphene and functional graphene using natural graphite as the starting material [86]. Among the different chemical methods, synthesis of solution processable graphite oxide as precursor of graphene-based materials is one of the most widely used protocols. In general, such methods involve chemical oxidation of natural graphite using various solution-based routes to synthesize hydrophilic graphite oxide followed by exfoliation (e.g., using ultrasonication) to form a single layer of few layer graphene oxide [87,88,89]. Thermal/chemical/electrochemical reduction of GO is normally used to produce reduced graphene rGO [86,90,91,92,93,94,95] (Figure 5).

**Table 1:** Different methods for synthesis of single or multilayer graphene.

Method	Precursor	Electronic quality	Advantage	Disadvantage	Commercialization	References
Mechanical Exfoliation	Graphite	High	Inexpensive and time saving method	Flakes randomly distributed, poor yield	Not scalable for commercialization	[76]
Arc discharge method	Graphite	High	Applicable to obtain Boron or nitrogen doped graphene	Cannot obtain pure graphene	Not scalable	[78]
Wet chemical synthesis such as Hummer, Brodie	Graphite	High	Transparent conductive film, useful to synthesis graphene based composites	Presence of oxygen impurities are not suitable for most of the electronic applications	Can be obtained in lab but not good enough for commercialization	[92]
Chemical vapour deposition	Hydrocarbons	High	Promising method that has all the above mentioned advantages	Transfer of graphene films deteriorates graphene quality and causes wrinkle formation	Possible	[80,82]
Solvothermal synthesis	Ethanol	Not available	Cheap and easily available precursor	Popcorn effect arises due to nucleation of sheets	Scalable	[96]
Epitaxial growth on metals	Ultrathin graphitic film	High	Single to multi layer graphene sheets can be obtained	Requires high temperature, expensive and difficult transfer process	Not feasible	[97]

During reduction GO, which is a non-conductive and hydrophilic layer of graphene with oxygen atoms attached to it, gets reduced to graphene. Currently, one of the most popular methods for synthesizing graphite oxide is Hummer’s or a modified Hummer method [86,94]. These methods for graphene preparation allowed facile large scale synthesis, and observation of its unique properties including: flexibility, transparency, chemical robustness and roll to roll processability. This protocol has the potential not only to significantly reduce the cost of mass-produced graphene, but also allow simple, non-chemical, thermal or electrochemical conversion of GO powder to graphene film or powder respectively. It has also widely opened the opportunity for a variety of functionalization and hybridization of graphene/few layer graphene and to tune the final characteristics. It is a potential method for synthesis of large-scale graphene to be used in various industrial applications including organic solar cells, capacitors, sensors, and transparent electrodes. The thermal reduction of GO to produce graphene; however, is a complex phenomenon and involves multistep removal processes of the intercalated H_2_O molecules, oxide groups of –COOH (carboxyl group), hydroxyl group and epoxy group. Therefore, the thermal reduction of GO and resultant GP needs to be investigated in great detail. Electrochemical reduction of GO has been successfully employed for graphene based electrochemical sensors and biosensors. Electrochemically reduced GO to rGO is the preferred method for reduction when rGO is to be used in different electrodes with different energy, electrochemical and electrochemical immunosensing platforms. [95,96,98,99,100] However, a much detailed understanding of the mechanisms of the exfoliation and reduction of graphite is essential to the design and discovery of efficient exfoliation systems with precise properties for specific applications.

## 6. Work Function and Tuning of the Work Function of Graphene

The work function, Ф of any material can be defined as the energy required to remove an electron from the highest filled level in the Fermi distribution of a solid to vacuum (*i.e.*, stationary at a point in a field-free zone just outside the solid) at absolute zero [97,101]:

Ф = *V*_vacuum_ − *E*_Fermi_ (1)

Work function (WF) is a fundamental electronic property of any material, and provides understanding of the relative position of the Fermi surface levels. WF, determines the band alignment in the contact at the heterojunction to facilitate selective electron and hole transport [101]. WF of graphene and the ability to engineer it, is a very important factor in governing the application of graphene as an electrode material [102]. Graphene is attractive as an important electrode material for transparent conducting electrodes in many modern optoelectronic devices including liquid crystal displays (LCDs), organic light emitting diodes (OLEDs), touch screens, and solar cells. Graphene is a zero band gap material, and its conductivity cannot be turned off electronically as in conventional semiconductor materials. It is a critical issue in graphene-based nanoelectronic and optoelectronic devices to tune the WF of graphene layers, while preserving its unique band structure [103]. For example, in organic thin-film devices such as light-emitting diodes (LEDs); the selection of an electron injecting contact with a WF that matches the energy level of the lowest unoccupied molecular orbital (LUMO) of the organic layer is critical. Such a choice prevents the formation of an electron injection barrier. Facilitating the charge injection improves the device performance, and tuning the WF of electrode to match the HOMO (or valence band) and/or LUMO (conducting band) of the active layers is essential [104,105]. Several physical and chemical methods including depositing the dopant atoms [106], absorption of gas molecules [107] or use of aromatic compounds [108,109,110,111] have recently been proposed for tuning the WR of graphene. Yu *et al.* [112] proposed tuning of graphene WR by electric field effect. Gui [113] proposed and modelled the band structure engineering of GR by application of strain. The chemical doping and deposition of different noble metal nanoparticles on the graphene surface is one of the most effective methods to tailor the WF of graphene [114,115]. Various chemical functionalization strategies also introduce band gap opening in graphene which leads to a change in work function [116]. The band gap opening leads to a shift in the Fermi level, however, the extent to which the band gap of graphene can be tuned has not been investigated in detail; and little is known about the precise role of interface and surface adsorbents [117]. In the following sections we will review in some detail different surface modifications techniques that have been used to modify graphene/GO/r-GO in an attempt to engineer the WF of graphene.

### 6.1. Effect of Oxygen Functionalities on the Work Function of Graphene

**Figure 6:**
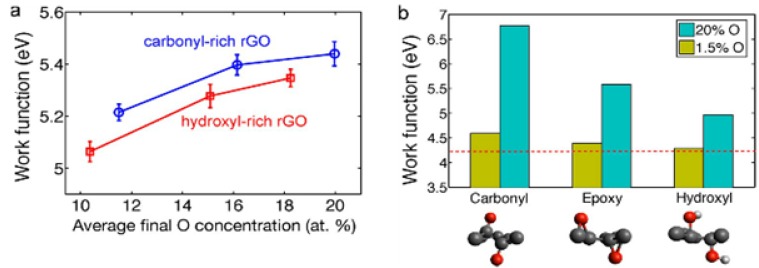
Work function tunability in rGO structures. (**a**) Calculated work function of carbonyl-rich and hydroxyl-rich rGO structures with different oxygen content; (**b**) The effect of individual functional groups on the work function of rGO, for two different total oxygen concentrations of 1.5% (for validation purpose) and of 20%. Change in Work function in rGO structures with respect to its functional groups. Reprinted with permission from [118]. Copyright 2013 American Chemical Society.

The wet chemical method based on oxidation of natural graphite to graphite oxide (GO) followed by exfoliation to graphene oxide, and finally thermal/chemical/electrochemical reduction of GO to reduced graphene oxide (r-GO) is one of the most widely used protocols for large scale synthesis of graphene/r-GO. This method, however, introduces different oxygen containing functional groups to graphene. The type and concentration of such functionalities have a decisive influence on the WF of graphene. For example, incorporation of electron withdrawing groups such as –OH to the surface of r-GO increases the WF; whereas, the addition of electron donating group (e.g., –CH_3_) to the surface decreases the WF [101]. Kumar *et al.* [118] recently thoroughly examined the impact of oxygen-containing chemical functionality on the WF of rGO using classical molecular dynamics simulation and density functional theory on a set of realistic rGO structures. From such an exercise, it was predicted that the presence of carbonyl groups on the rGO surface results in the largest impact on WF (6.7 eV) relative to all other groups. It has been demonstrated that the presence of such a group can induce a WF value of ~60% higher than that of pristine graphene (4.2 eV). On that basis they projected a significant tunability in the WF of rGO (up to ~2.5 eV) by altering the composition of the oxygen-containing functional groups (Figure 6). Mishra *et al.* [119] experimentally evaluated the WF of GO as a function of oxygen content. They employed contact surface potential difference (CPD) estimation as an indicator of WF using a scanning Kelvin probe method (SKPM). SKPM, is a scanning probe method, where, a vibrating capacitor is used to investigate the WF of metal and semiconductor surfaces at nano/micro level; and provides information about the electronic state of the local structures on the surface of a solid. In principle it is based on the detection and dynamic compensation of the electrostatic forces that arise between the micro/nano tip and the surface when they are electrically connected [119]. CPD measurement estimated WF indirectly in terms of relative surface contact and may be converted to an absolute value by the appropriate calibration. The CPD measurement between two different surfaces follows the relation:

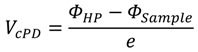
(2)
where Φ is the WF, and *e* is the elementary charge of electron [119].

**Figure 7:**
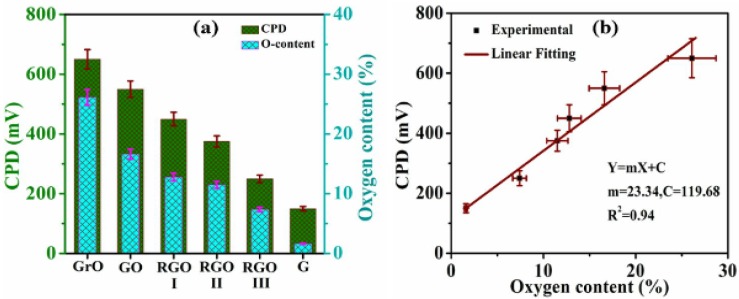
(**a**) Bar graph of contact surface potential difference (CPD) as a function of oxygen content at different stages in graphene synthesis; and (**b**) Linear fitting of CPD variation during decreasing oxygen content at different stages from graphite oxide to graphene. Reprinted with permission from [119]. Copyright 2013 American Chemical Society.

Figure 7 illustrates the variation of experimental CPD as a function of oxygen content for Go to r-GO. It is clear from Figure 7b that CPD and oxygen content at different stages from GO follow a linear trend. These recent predictive methods and experimental study demonstrated the possibility of precise control of WF of rGO by tuning the oxygen-containing functionalities on the surface. However, further in-depth experimental investigation of the effect of different type and concentration of oxygen containing functionality is critical to fine tune the WF of graphene.

### 6.2. Work Function Engineering by Reduction of Graphene Oxide

**Figure 8:**
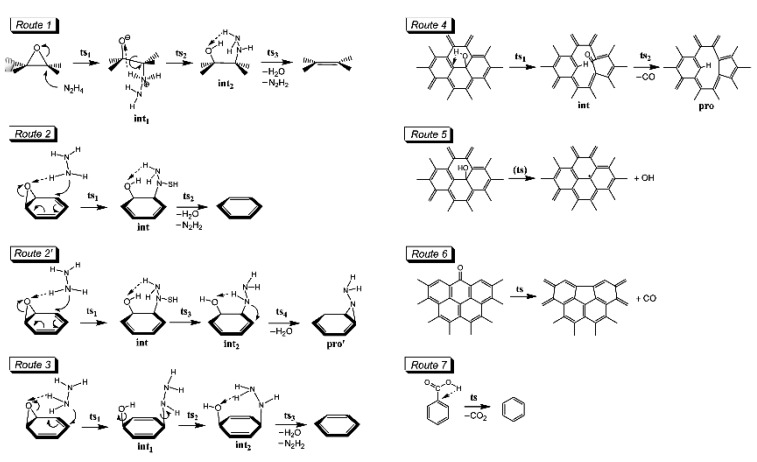
GO reduction mechanism. Routes1–3 and 2’represents the mechanism for hydrazine de-epoxidation of GO. Routes 4–5 represent the mechanism for thermal dehydroxylation for GO. Routes 6 and 7 represent the mechanism for thermal decarbonylation and thermal decarboxylation of GO. Reprinted with permission from [128]. Copyright 2009 American Chemical Society.

An extensive amount of research effort has been focused on thermal [120,121], chemical [14,122,123], and electrochemical [124,125] reduction of solution processed GO to grapheme [126,127]. Reduction of graphene is nothing but removal of oxygen functionalities present on the GO surface, which will ultimately alter the WF of the rGO. However, actual chemical transformation during the reduction process is complicated and significantly dependent on the method used for reduction and the environmental conditions imposed during the reaction. Different reduction methods have different effects on the graphene surface, and consequently result in different WFs. As the presence of surface functionality is the most important factor to control WF of graphene; understanding the reduction mechanism for removal of different functional groups at different steps is very important. Gao *et al.* [128] reported the reduction of GO through simultaneous chemical and thermal reduction methods. They elucidated the mechanism of chemical reduction via hydrazine as decarbonylation, and thermal reduction at 900 °C as dehydroxylation of GO (Figure 8). Higher C to O ratio was observed as GO was progressively annealed from 500 to 900 °C.

**Figure 9:**
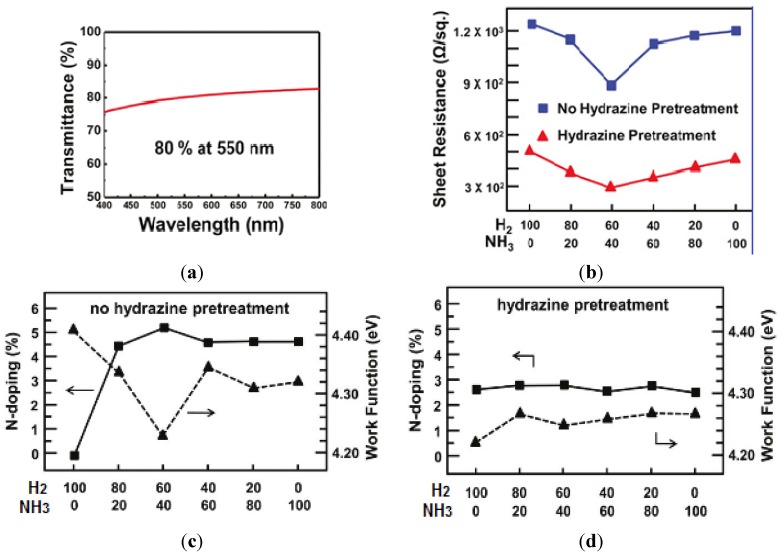
Effect of GO treatment on the properties of GO. (**a**) Shows increase in transmittance with respect to wavelength and (**b**) Shows decrease in sheet resistance with respect to hydrazine treatment, whereas (**c**) Shows percentage of N-doping and work function modulation with no hydrazine pre-treatment, and (**d**) With hydrazine pre-treatment plotted again H_2_/NH_3_ ratio. Reprinted with permission from [129]. Copyright 2011 American Chemical Society.

Hwang *et al.* [129] attempted to tune the WF of N-doped rGO film. N-doped reduced graphene was obtained by sequential chemical treatment of GO with hydrazine followed by thermal treatment. N-doping was performed to increase the electron density and thus further reduce WF. They observed that the hydrazine treatment lowered the oxygen containing groups; whereas, the thermal treatment removed the less stable N and O containing groups present at dangling bonds. The WF for N-doped graphene, reduced using hydrazine based pre-treatment, showed almost a constant value of WF (4.25 eV). However, N-doped films without pre-treatment exhibited variation in WF value within the range of 4.2–4.4 eV; and such rGOs exhibit higher conductivity, superior transmittance (~80%) and lower sheet resistance (Figure 9). Hydrothermal reduction techniques have been employed by Chieh *et al.* [130], to tune the WF of GO over a wide range from 5.72 to 4.43 eV. They examined the treated GO using X-ray absorption near edge structure (XANES), and confirmed progressive increase in the sp^2^ to sp^3^ ratio with respect to increase in the hydrothermal temperature. This observation is consistent with the increase in conductivity with increased amount of sp^2^ species with a progressively higher treatment temperature of GO. The use of a combination of treatments provides opportunity to fine tune the WF for graphene for many applications including cathode material, to obtain highly efficient polymer light emitting diodes (PLEDs).

Liu *et al.* [131] fabricated a polymer solar cell using graphene as the hole transport layer (HTL) and P3HT(donor)/PCBM (acceptor) as active layer, and the reported *V*_OC_ was 0.6 V at 130 °C. However, they observed a decrease in open circuit voltage (*V*_OC_) when GO was annealed to 230 °C. The *V*_OC_ value of a device is governed by the difference in WF between HOMO of the donor (P3HT (5.0 eV)) and LUMO of the acceptor (PCBM (3.7 eV)) material. The authors ascribed this change in *V*_OC_ with thermal treatment and the WF change in GO due to the removal of functional groups at higher temperature.

### 6.3. Work Function Engineering of GO Using Functionalization and Self-Assembled Monolayer

**Figure 10:**
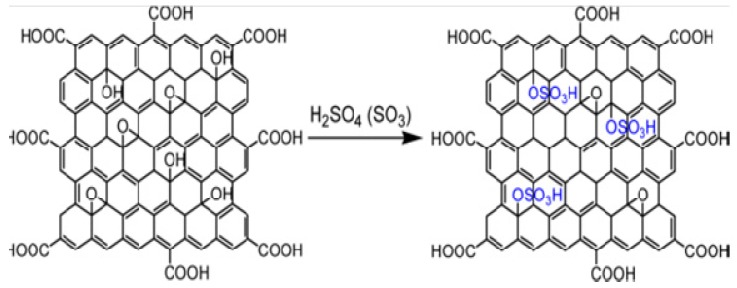
Formation of GO–OSO_3_H. Reprinted with permission from [132]. Copyright 2012 American Chemical Society.

Functionalization of GO or graphene using various chemical approaches has been attempted to modulate WF [109]. Liu *et al.* [132] reported tuning of the WF of GO to match the WF of higher occupied molecular orbital (HOMO) of P3HT (5.0 eV) through synthesis of sulfated graphene oxide (GO–OSO_3_H), and achieved a WF of 4.8 eV. To achieve such a goal, GO was functionalized (Figure 10) where the –OSO_3_H group was attached to the –COOH groups present at the edge of GO. Such WF matching has the potential to enhance the conductivity of the charge transfer layer of organic solar cells. Yang *et al.* [133] demonstrated O_2_ plasma treatment of GO to achieve a work function of 5.2 eV. Such chemically modified GO could be used as a hole transport layer in organic solar cells.

By considering the influence of functional groups and strong pi-pi conjugation (that leads to changes in Fermi level) between graphene and P3HT on WF, Wang *et al.* [134] attempted to tune the WR of graphene with a view to use solution-processable functionalized graphene as the acceptor material in OPV to replace PCBM (e.g., Phenyl-C61-butyric acid methyl ester). They used phenyl isocyanate-treated GO to obtain SPF-graphene to replace acceptor component. SPF-graphene can be easily dissolved in organic solvents such as 1,2-dichlorobenzene (DCB), which is also the solvent for P3HT. Such photovoltaic devices can be fabricated using common fabrication processes, and the active layer can be prepared by spin coating a P_3_HT/SPF graphene solution in DCB. The WF difference between graphene and P_3_HT is generally ~0.7 eV, which was further increased to 1.08 eV for SPF-graphene. The investigators confirmed the influence of WF on *V*_OC_ by subjecting SPF-graphene/P_3_HT active layer to a range of annealing temperatures. They demonstrated that the *V*_OC_ value of the system was between 0.7 and 1.1 eV on annealing the system at 160 °C, whereas the value fell to 0.5 eV when the annealing temperature was increased to 210 °C. This change in WF was attributed to the elimination of functional groups at higher temperature, which led to a change in WF and thus *V*_OC_ [122]. Similar observation was reported by Wang *et al.* [135] when SPF-graphene was used as acceptor along with PCBM and P3OT as donor.

Liu *et al.* [136] fabricated an OPV device with P3HT/SPF-graphene as active layer; and they reported a short circuit current density *J*_sc_ of 4.0 mA·cm^−2^, open circuit voltage *V*_OC_ of 0.72 V, and a solar power conversion efficiency of 1.1% under simulated AM1.5G, 100 mW illumination in air for such a device. This observation indicates that soluble graphene has the potential to be a promising acceptor material for OPVs; however, further research is critical to optimise the donor/acceptor interface engineering and work function tuning. Recently, Lin *et al.* [137] further reported findings, both theoretical and experimental, on the graphene/semiconductor heterojunction solar cells. They predicted that for such a Schottky junction solar cell a maximum theoretical conversion efficiency of ~9.2% could be achieved by controlling the graphene layer number, tuning the graphene WF and adding an antireflection film. They also experimentally demonstrated that solar cells based on modified graphene and Si pillar arrays can deliver enhanced cell performance with efficiencies of up to 7.7%.

### 6.4. Work Function Engineering Using Self-Assembled Monolayer (SAM) and Layer by Layer Technique

**Figure 11:**
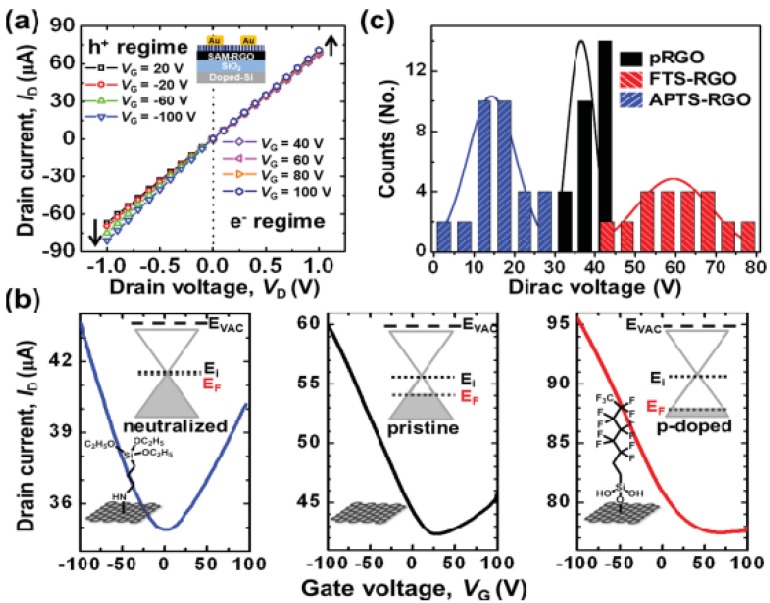
Work functions of graphene can be widely tuned using direct surface functionalization, which is demonstrated by self-assembled monolayers anchored onto the surfaces of the r-GO. Charge-transport characteristics of r-GO field-effect transistors (FETs) functionalized with the various self-assembled monolayers (SAMs). The inset of (**a**) shows the device configuration, where r-GO was used as an active layer. (**a**) Output characteristics of pristine r-GO FETs; (**b**) Transfer characteristics (*V* D = −1 V) of various r-GO FETs: APTS-rGO (**left**), pr-GO (**middle**), and FTS-r-GO (**right**). The insets show schematic band diagrams of SAM-functionalized r-GOs; (**c**) Comparative plots of Dirac voltages of the r-GO FETs. Reprinted with permission from [138]. Copyright 2013 Wiley-VCH.

Kang *et al.* [138] investigated the influence of the tuning of the WF of rGO by self-assembled monolayer (SAM) formation on p-channel organic field-effect transistors (FETs). They showed that the use of (tridecafluoro-1,1,2,2-tetrahydrooctyl) trichlorosilane SAM modified GO (FTS-GO) display strong p-doped behavior relative to r-GO. FTS-rGO exhibited WF of 5.51 eV, a considerable increase when compared to the WF for r-GO of 4.9 eV. However, aminopropyl triethoxysilane (APTS) SAM modified rGO (APTS-rGO), exhibited WF of 4.31 eV, which is 0.63 eV lower than that of p-RGO (Figure 11). This tunability of WF for SAM functionalized rGO was utilized in developing source/drain electrodes in bottom-contact FETs. It is suggested that the APTS functionalization helps in neutralization of unintentionally doped p-rGO [138]. Layer-by-layer technique is a facile means of fabricating multilayer hybrid thin films. Kong *et al.* [139] employed LBL assembly to make a hybrid thin film of graphene and gold nanoparticles. They demonstrated that for such films electrons in the negatively charged (rGO) substrate participated in the reduction of metal (Au) cations. Such electrochemical transformation occurs because the reduction potential of rGO is much less than that of Au (+1.002 V). The significant variation in reduction potential allows reduction of Au cations by donating electrons from rGO to Au^3+^. This material was found to be highly applicable to sensors and other electronic devices.

### 6.5. Work Function Engineering of Graphene Using Noble Metal Doping

The band gap structure, and the type and the concentration of carriers in graphene—both electrons and holes—can be controlled by the introduction of metals or molecules on the graphene surface. This is easily possible because, zero-gap 2D semiconductor graphene has extreme sensitivity to molecular adsorbents. The relative position of the HOMO and LUMO of the adsorbents with respect to the Dirac point in pristine graphene determines the direction of charge transfer. In general GO and r-GOs synthesized by oxidation and reducing exfoliated graphite possess many defects including vacancies, oxide functionalities and substitutional nitrogen formed by harsh chemical treatments. The presence of these defects and the functionalities perturbs the π–π network of graphene, resulting in a loss of electrical conductivity of the GO/r-GO structure. However, it has been identified that GO/r-GO has the potential to recover conductivity through molecular doping by defect filling. Moreover, the presence of various oxygen containing functional groups on GO and rGO make them suitable platforms for the nucleation and growth of noble metal nanostructures, and synthesis of noble metal nanoparticle/graphene composites [140,141].

The controlled synthesis of hetero-structures based on Ag, Au, Pt with graphene has attracted significant attention in recent years due to the unique size and shape dependent properties including high catalytic activity, resulting in potential applications in chemical sensors, energy storage devices, catalysis, hydrogen storage, fuel cells, solar cells, electrochemical sensors and surface enhanced Raman scattering [140,141,142,143,144,145,146,147,148,149,150]. Huang *et al.* [127] recently presented a review on the current development of graphene-based composites including metal-graphene composites. Subrahmanyam *et al.* [151] examined the specific interaction of nanoparticles of metals such as Ag, Au, Pt and Pd with graphene using Raman spectroscopy and first-principles calculations. A significant charge-transfer interaction between graphene and the metal nanoparticles was demonstrated through both experimental and theoretical calculations. Lightcap and Kamat [152] discussed the potential of the graphene/semiconductor and graphene/metal-nanoparticle composites to function as efficient, multifunctional materials for energy conversion and storage. They emphasized that such advanced composites have the promise to integrate conversion and storage of solar energy, the detection and selective destruction of trace environmental contaminants, and to achieve single-substrate, multistep heterogeneous catalysis. In the next section, we attempt to discuss in some details various approaches used for tuning the WF of graphene-based noble metal nanostructures that can be useful as both electroactive and photoactive components in many devices.

### 6.6. Work Function Engineering on Graphene Based Gold (Au) Composite

**Figure 12:**
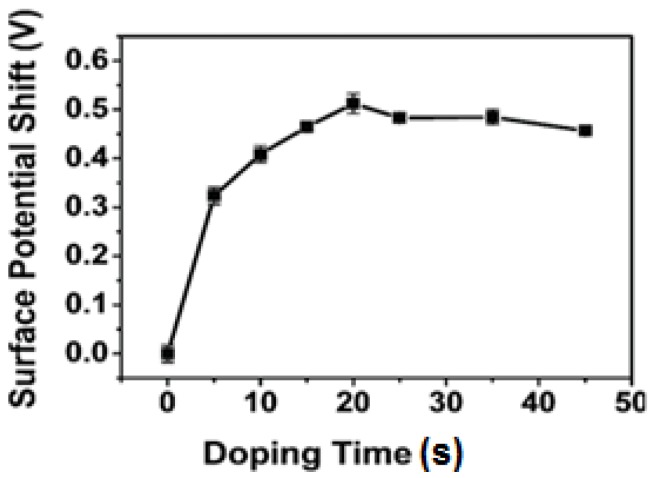
Change in WF with respect to doping time. Reprinted with permission from [60]. Copyright 2010 American Chemical Society.

**Figure 13:**
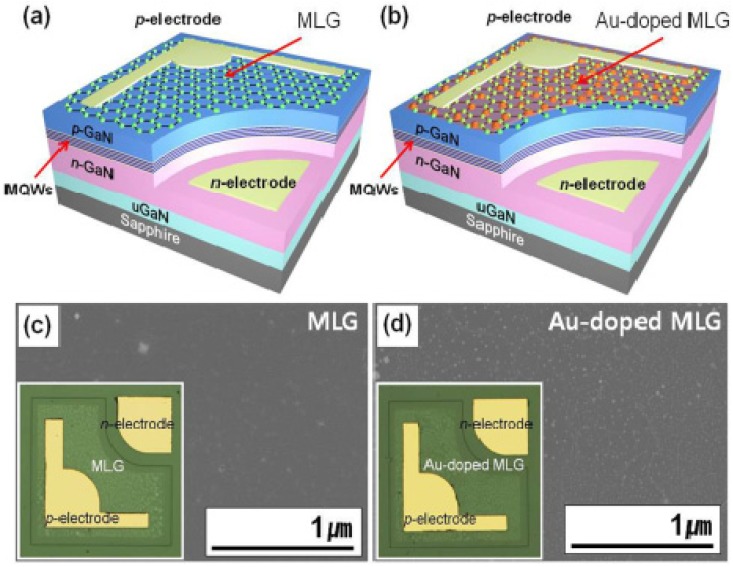
Schematic diagrams of near-ultraviolet light-emitting diodes (NUV-LEDs) with (**a**) multi-layer graphene (MLG) and (**b**) Au-doped MLG TCLs; (**c**) SEM images of MLG and (**d**) Au-doped MLG. Reprinted with permission from [155]. Copyright 2013 AIP Publishing.

Graphene is a promising nanoscale building block of new nanocomposites and can act as a support material for the dispersion of metal nanoparticles. Kim *et al.* [153] reported AuCl_3_-doped graphene transparent conductive electrodes that were integrated in GaN-based ultraviolet (UV) light-emitting diodes (LEDs). They reported that the transmittance and the sheet resistance of the graphene electrode layer decreased with increase in the concentration of the AuCl_3_ in solution. It was also demonstrated that *p*-type doping of the graphene by AuCl_3_ dramatically improved the I-V characteristics and EL intensities. However, there is an optimal in the level of AuCl_3_ due to the trade-off between the decrease in both transmittance and the sheet resistance of the graphene electrode with increase in AuCl_3_. Choe *et al.* [154] investigated the WF of AuCl_3_-doped graphene layers, and reported that the WF of p-doped graphene layers exposed to 20 mM AuCl_3_ solutions increased from 4.42 to 5.12 eV. The p-doping occurs due to the electron transfer from the graphene layer to the Au nanoparticles. Shi *et al.* [60] also examined surface modification of graphene using Au dopant (e.g., AuCl_3_) for specific range and demonstrated the upshift in WF of graphene film to ~0.5 eV (Figure 12). Cho *et al.* [155] demonstrated the efficiency of gold (Au) doped multi-layer graphene (MLG)/AuNP composite as a transparent conducting layer in near-ultraviolet light-emitting diodes (NUV-LEDs) (Figure 13). They reported that the use of thermally annealed Au-doped MLG enhanced the optical output power of NUV-LEDs by 34% relative to that of NUV-LEDs with a bare MLG. Au-doped MLG exhibited low sheet resistance and high current injection in the NUV-LED. A shift in WR of Au doped GR from 4.5 eV (undoped) to 4.9 eV (doped) was demonstrated, and a decrease in sheet resistance (*R*s) from 500 to 90 Ω^−2^ was observed. The improved I-V characteristics of the NUV-LEDs with an Au-doped MLG layer can be attributed to the reduced sheet resistance of the Au-doped MLG films, and the decreased contact resistance between Au-doped MLG and the p-GaN layer.

**Figure 14:**
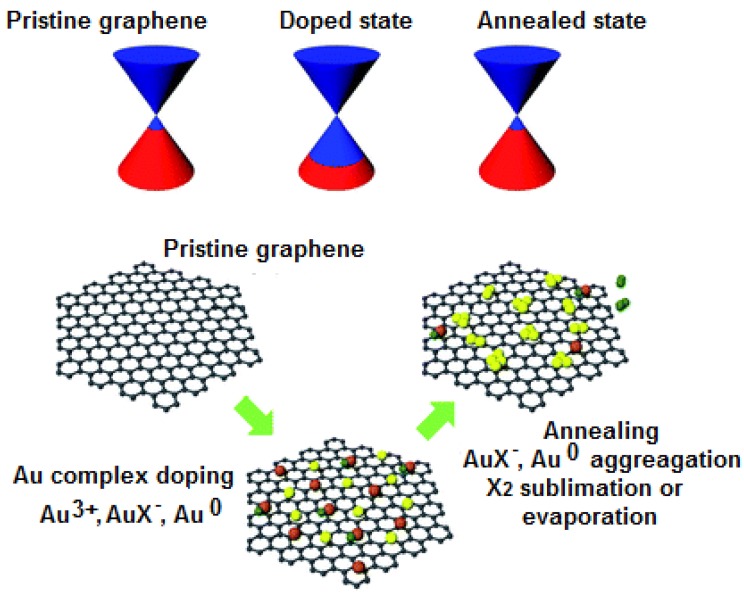
Schematic representation of the Dirac point state and morphological state of graphene according to sample treatment condition. Schematic representation of morphological state of graphene, before treatment, after treatment and after annealing are shown (left to right). Reprinted with permission from [156]. Copyright 2013 Royal Society of Chemistry.

Kwon *et al.* [156] tuned the WF of Au/graphene composite using different Au precursors containing different anions. They obtained WF of 4.3–4.6; 4.8; 5.0; 4.9 eV when Au (OH)_3_; Au_2_S; AuBr_3_; AuCl_3_ respectively were used as the precursors (Figure 14). They also demonstrated that thermal annealing of the doped graphene surface decreases the WF (Figure 14b). The decrease in WF value was attributed to the breakage of ionic bonding between Au cation and anion in each dopant doped cationic graphene. This observation indicates the sensitivity of the WF on surface engineering. Goncalves *et al.* [140] examined the role of oxygen moieties at GO and rGO surfaces on the gold nucleation and growth. They reported that the nucleation and growth mechanism depends strongly on the degree of oxygen functionalization on the graphene surface. No AuNP was reported to be observed at totally reduced graphene surfaces.

**Figure 15:**
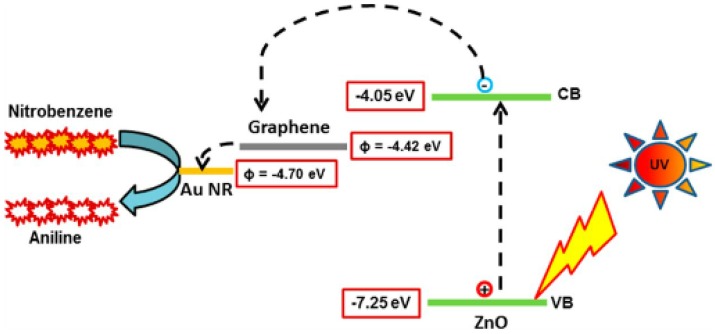
Schematic diagram showing the electron transfer mechanism from different energy levels of the Graphene-ZnO-Au heterostructure for the photo-reduction of nitrobenzene. Reprinted with permission from [146]. Copyright 2013 American Chemical Society.

A facile fabrication of macroporous gold films using graphene sheets as template was reported by Sun and Wu [157]. AuNP decorated graphene sheets were prepared using a one-pot simultaneous reduction of GO and gold precursor HAuCl_4_ by sodium citrate. Roy *et al.* [146] recently reported a highly efficient photocatalytic graphene-ZnO-Au nanocomposite using a simple hydrothermal method. This type of complex nano-composite has great potential in removing organic pollutants such as nitrobenzene. A rapid conversion of nitrobenzene to aniline within 60 s was demonstrated, and it was interpreted on the basis of WF tuning and electron-hole combination. It was implied that WF decreased via rapid electron transfer from ZnO to graphene (WFs of conduction and valence bands of ZnO are −4.05 eV and −7.25 eV respectively, and WF for graphene and Au are −4.42 eV and −4.70 eV) (Figure 15). It was hypothesised that the presence of solvent such as methanol (hydroxyl group) contributes to trapping holes, enables photo-generated electrons to catalyse reduction of nitrobenzene (NB) to aniline with a yield of 97.8%. Koo *et al.* [158] observed the growth of sub-nano sized Au clusters on r-GOs, which could reinforce the conductivity of the resulting r-GOs by defect filling. The resulting Au/r-GOs were reported to exhibit an improvement of bulk electrical conductivities and a reduced ratio of the intensity of the D band to that of the G band (ID/IG), relative to the r-GOs without Au nanoclusters. The decrease of the ID/IG was explained to be related to the filling of sub-nano sized Au clusters on the r-GOs owing to the enhancement of the flat geometry of the graphene nanosheets.

### 6.7. Work Function Engineering on Graphene Based Silver (Ag) Composite

For the synthesis of Ag-graphene nanostructure, AgNO_3_ is the commonly used precursor, which can be easily reduced in the presence of different reducing agents including amines, NaBH_4_ and ascorbic acid. [159,160]. Dutta *et al.* [161] reported synthesis of Ag-nanoparticle conjugated rGO nanosheets using dimethylformamide (DMF) as an efficient reducing agent that reduces both silver nitrate (AgNO_3_) and graphene oxide (GO) in the reaction mixture. Shim *et al.* [162] demonstrated that in the case of GaN-based LEDs the WF of graphene could be tuned from 4.3 to 5.0 eV by introducing Ag, and such a device could produce uniform and stable light emission: 1.7 times higher than graphene only LEDs. Recently, the importance of WF was revealed during electron transfer from a Rhodamine B (RhB) dye to Ag/Graphene nanocomposites by Martínez-Orozco [163]. RhB (5.67 eV), after exciting electron, was converted to RhB* (3.08 eV) that acted as sensitizer in visible light. It was highlighted that RhB* injected electrons to the electron acceptor, *i.e.*, graphene (4.26 eV) to become a cationic radical RhB^+^; which underwent self-degradation by trapping excited electrons of adsorbed oxygen. During this process, recombination of the injected electron and surface adsorbed RhB^+^ might be possible, however, it was hypothesised it could be stopped by transferring injected electrons from graphene to Ag-nanoparticles leading to a better separation of electron (e^−^) and hole (h^+^). This electron transfer process that occurs on the basis of WF difference proves the importance of WF study in energy conversion devices.

### 6.8. Work Function Engineering for Graphene Based Platinum (Pt) Composite

Pt nanoparticles supported on a highly dispersed support have attracted much attention, and this is one of the most widely used catalyst materials for many chemical reactions and energy conversion devices including hydrogen oxidation and oxygen reduction reactions in fuel cell. Thus, significant efforts have been focused to combine graphene as a conductive support for Pt nanostructures. The surface area of graphene is exceptionally high and has also the potential to reduce carbon corrosion resulting in enhanced electrocatalytic performance and long term stability. Most of the techniques that were used to prepare graphene-Au or Ag composites and discussed above could be equally applied in general to fabricate graphene supported Pt nanomaterials. K_2_PtCl_4_ has been commonly used as a precursor that can be reduced on the surface of graphene to form Pt NPs [143,149,164,165,166]. Li *et al.* [165] synthesized Pt-nanoflowers loaded on rGO using a green approach for methanol electro-oxidation. Graphene supported Pt nanostructures have been synthesized that can be used as electrodes in fuel cells or other energy related devices [149,165,166,167]. In rGO-NP heterostructures, the electronic structure of GO/rGO is preserved by weak adsorption on these metals. However, even when the bonding is weak, the metal substrates caused the Fermi level of graphene to move away from the conical points, resulting in doping with either electrons or holes. The amount of doping in any specific case is dependent on the difference in WF of metal and graphene. Therefore, tuning of WF can be performed by doping graphene using different metal substrates such as Au, Ag, Pt, *etc.*, and a shift of WF has been observed from 5.54 to 4.74 eV, 4.92–4.24 eV, and 6.13–4.8 eV, respectively [61]. It is realized that graphene-supported noble metal nanostructures are promising electrodes and photoactive material for energy conversion and sensing applications [168]. However, significant further fundamental investigation on the effect of quality of interaction between the support and the NP on the WF is essential to make graphene a multipurpose material for energy conversion and sensing devices. Table 2 illustrates WF data for graphene based noble metal composites using a variety of precursors and their respective advantages and applications.

**Table 2:** Work function data for graphene based noble metal composites.

S.No.	Modified Graphene	Method	Precursor	WF (eV)	Improved property	Application	Ref.
1	Graphene-ZnO-Au	Hydrothermal	Zn Acetate , HAuCl_4_	Value not given	Efficiency = 3.5–4.5 fold more than ZnO	Removing pollutant (nitrobenzene)	[157]
2	Various Au dopants on graphene	Chemical doping	AuBr_3_, Au_2_S, Au(OH)_3_, AuCl_3_	5, 4.8, 4.6, 4.9 as doped and 4.5, 4.4, 4.55, 4.3 eV as annealed (w.r.t. the precursor)	Multiuse of graphene due to tunable WF property	Energy conversion devices and sensors	[159]
3	Au/graphene	Chemical doping	AuCl_3_	Increase by 0.5 eV with increase in doping time	Tunable WF property	Optoelectronic devices	[60]
4	Ag/graphene	Photochemical silver functionalization	AgNO_3_	Value not given	Suppressed e-h recombination	Efficient removal of hazardous materials	[161]
5	Au/Ag/Pt-graphene	Graphene adsorption metal substrate	Au, Ag, Pt substrates were used	5.54–4.74, 4.92–4.24, 6.13–4.8 eV	Multipurpose modified graphene	Energy conversion devices	[168]

## 7. Conclusions

High-quality graphene has only been available for less than a decade; however, it has generated unprecedented excitement in the scientific community. The electronic structure, unique morphological and electronic characteristics of graphene and its potential applications have been the subject of extensive theoretical and experimental investigations. This advanced material has not only afforded the Nobel Prize to Andrei Geim and Konstantin Novoselov in 2010 “*for ground-breaking experiments regarding the two-dimensional material graphene*” but has also generated great interest to wide ranging industries including semiconductor, optoelectronics and printed electronics. Graphene has indeed been identified as a next-generation material with potential to replace traditional electrode materials in many electronic and optoelectronic devices. One of the most important themes of current research on graphene is to understand the interaction between graphene and its surrounding environment including the presence of optically and electrochemically active nanoparticles, and the consequences on the band gap structure and Fermi level. Such investigations are of significant fundamental interest from the quantum physics point of view, as well as being relevant for the process of sample production and fabrication of graphene-based electronic and optoelectronic devices. The influence of the presence of ad atoms, different functionalities and modifications on the WF of graphene, GO and r-GO has been studied to understand the different functionalization strategies to tune the WF of graphene. This effect of WF modulation has proved to be very useful in employing graphene as a multipurpose material for applications related to energy conversion devices such as an electrode, hole transporting layer (HTL), photoactive material. The surface functionalization of solution processable graphene has attracted significant attention as a potential method for the synthesis of graphene to be used in various industrial applications including photovoltaic cells, capacitors, sensors, and transparent electrodes. Recent innovations in the continuous and scalable large area synthesis of graphene using chemical vapour deposition (CVD) have reinforced its study in a broad range of research areas. The most critical technological challenge to be faced before a wider application of graphene is to develop a facile and efficient method for controlled production of processable single/few layer large graphene sheets of the desired properties, and tuning of the electronic properties including work function.
